# Prediction of lymphedema occurrence in patients with breast cancer using the optimized combination of ensemble learning algorithm and feature selection

**DOI:** 10.1186/s12911-022-01937-z

**Published:** 2022-07-25

**Authors:** Anaram Yaghoobi Notash, Aidin Yaghoobi Notash, Zahra Omidi, Shahpar Haghighat

**Affiliations:** 1 The Computer Engineering Department, Rasht Branch, Islamic Azad University, Rasht, Iran; 2 Shariati Hospital, Tehran University of Medical Science (TUMS), Tehran, Iran; 3 Breast Cancer Research Center, Motamed Cancer Institute, ACECR, Tehran, Iran

**Keywords:** Breast cancer, Lymphedema, Data mining, Ensemble learning, Feature selection

## Abstract

**Background:**

Breast cancer-related lymphedema is one of the most important complications that adversely affect patients' quality of life. Lymphedema can be managed if its risk factors are known and can be modified. This study aimed to select an appropriate model to predict the risk of lymphedema and determine the factors affecting lymphedema.

**Method:**

This study was conducted on data of 970 breast cancer patients with lymphedema referred to a lymphedema clinic. This study was designed in two phases: developing an appropriate model to predict the risk of lymphedema and identifying the risk factors. The first phase included data preprocessing, optimizing feature selection for each base learner by the Genetic algorithm, optimizing the combined ensemble learning method, and estimating fitness function for evaluating an appropriate model. In the second phase, the influential variables were assessed and introduced based on the average number of variables in the output of the proposed algorithm.

**Result:**

Once the sensitivity and accuracy of the algorithms were evaluated and compared, the Support Vector Machine algorithm showed the highest sensitivity and was found to be the superior model for predicting lymphedema. Meanwhile, the combined method had an accuracy coefficient of 91%. The extracted significant features in the proposed model were the number of lymph nodes to the number of removed lymph nodes ratio (68%), feeling of heaviness (67%), limited range of motion in the affected limb (65%), the number of the removed lymph nodes ( 64%), receiving radiotherapy (63%), misalignment of the dominant and the involved limb (62%), presence of fibrotic tissue (62%), type of surgery (62%), tingling sensation (62%), the number of the involved lymph nodes (61%), body mass index (61%), the number of chemotherapy sessions (60%), age (58%), limb injury (53%), chemotherapy regimen (53%), and occupation (50%).

**Conclusion:**

Applying a combination of ensemble learning approach with the selected classification algorithms, feature selection, and optimization by Genetic algorithm, Lymphedema can be predicted with appropriate accuracy. Developing applications by effective variables to determine the risk of lymphedema can help lymphedema clinics choose the proper preventive and therapeutic method.

## Introduction

Breast cancer accounted for 12% of all cancer diagnoses, 6.6% of cancer deaths, and 2.09 million cases in 2018, making it the most common cancer among women [1, 2]. The incidence of this cancer is expected to increase, and the number of patients will increase to 2.1 million by 2030 [3]. Therefore, early detection and improved survival are essential [4].

Breast cancer-related lymphedema is one of the most common disorders following breast surgery, radiation therapy, and chemotherapy, seen in one of five patients with breast cancer [5]. Treatments for breast cancer and other risk factors such as obesity, decreased activity, and limb injuries lead to anatomical defects in the lymphatic system and put individuals at risk of lymphedema [6].

Through the progressive accumulation of lymph fluid in the interstitial tissue space, lymphedema leads to persistent swelling in the affected arm, shoulder, neck, breast, or injured area. It can reduce patients' quality of life by psychological and physical consequences such as anxiety, depression, adjustment problems, social and sexual problems, reduced range of motion of joints, pain, arm heaviness, skin changes, fibrosis, and cellulite [6, 7]. None of the available pharmacological and non-pharmacological treatments is considered the definitive treatment of lymphedema, nor do they reduce its severity [8]. Lymphedema is diagnosed and evaluated clinically by observing swelling or other signs, circumferential measurements in centimeters, and volumetric measurements in milliliters. However, these methods are used for patients with clinical lymphedema, so an accurate therapeutic outcome may not be achieved at this stage [9].

For this reason, lymphedema risk factors are currently being discussed more to prevent this complication by recognizing the risk factors and performing effective and timely interventions. Early and continuous treatment significantly slows the progression of the disease and reduces tissue damage. The sooner the treatment is started, the better the chance of recovery [10]. Studies have identified the following as the most significant risk factors: lymph node dissection, mastectomy, high body mass index (BMI), number of the involved lymph nodes, lack of regular physical activity, receiving chemotherapy and radiation therapy [11], however, determining these risk factors in different populations can provide appropriate interventions for the same population. Therefore, developing a model to predict the likelihood of this complication is necessary. With digital technologies at the service of public health, therapeutic outcomes in breast cancer survivors can be improved through methods such as data mining algorithms, a powerful tool for predicting and determining the risk factors of lymphedema.

Data mining, a part of decision sciences, is a technique for evaluating information. It can be used in decision making, forecasting, prediction, and estimating the consequences. Medical data mining has a great potential for discovering hidden patterns in data [12]. Some studies have examined the application of data mining algorithms in chronic diseases. A study by Qasem Ahmad evaluated data mining models for predicting breast cancer recurrence in 547 patients. It showed that the affecting variables of the disease recurrence were degree of lymph node involvement, tumor size, and lymph node inflammation. The SVM classification model has a higher sensitivity and specificity in predicting breast cancer recurrence [13]. A data mining technique was used to predict myocardial infarction in another study. The variables of hypertension, hyperlipidemia, and smoking were risk factors and predictors of myocardial infarction [12].

Other studies compared the performance of five machine learning algorithms in detecting lymphedema and reported that the artificial neural network achieved the best performance with an accuracy of 93.75% [9]. Another study included a database for patients from June 2018 to June 2019 in the Iran Cancer Research Center. Their results show that the C5.0 algorithm could be an acceptable tool for predicting lymphedema occurrence [14]. A study for predicting kidney stones proposed the ensemble learning method. In this study, weight was assigned based on the Genetic algorithm-based model with an accuracy of 97.1%. Finding the optimal weight vector and adjusting its properties is a fundamental issue [15]. Some studies have proposed new frameworks for an intrusion detection system based on selected features and techniques of collective learning with a set of different classes [16, 17]. Improving breast cancer diagnosis and treatment can increase the number of survivors, so determining risk factors of breast cancer-related lymphedema and predicting it can improve patients' quality of life. This study aimed to assess an appropriate model to predict the risk of lymphedema and determine the effective and predictive factors based on a more appropriate model.

## Materials and methods

This study is cross-sectional research on data from patients with breast cancer-related lymphedema through a data mining algorithm. The database was collected in the Seyed_Khandan lymphedema clinic from 2009 to 2018 and included data from 1117 patients. This clinic is one of Iran's cohesive and focused lymphedema centers in providing diagnostic and treatment services. Therefore, the obtained data can be generalized to other countries' lymphedema patients. In this clinic, all patients' demographic characteristics, clinical data, and edema volume during the first visit are registered in the SPSS software. Most of the recorded variables are considered lymphedema risk factors in the current study. The data of each patient was recorded and analyzed separately via the MATLAB draft commands, and the base data mining algorithms processed the input data.

To achieve the study goals, the steps of determining an appropriate model to predict the risk of lymphedema and identifying the effective factors, two separate phases were conducted as follows:

### Phase 1: determining the appropriate model to predict the risk of lymphedema

#### Data preprocessing methods

At first, the best single algorithm used to classify the data was determined. The obtained data included 54 variables in three groups of demographic, clinical, and lymphedema-specific characteristics as follows:

- Demographic characteristics: name, date of birth, height, weight, occupation, dominant hand, education, marital status, place of residence, and area involved (according to the patient).
- Clinical characteristics: type of biopsy, tumor size, sentinel lymph node biopsy, number of removed lymph nodes, number of involved lymph nodes, stage of the disease, estrogen and progesterone receptor status, presence or absence of metastasis, diagnosis date, type of surgery, date of surgery, chemotherapy, chemotherapy regimen, number of chemotherapy sessions, radiotherapy, hormone therapy, receiving Herceptin, comorbidities, physical activity, and chief complain at the first visit.
- Lymphedema-specific characteristics: feeling of heaviness, paresthesia, history of limb injury, infection history, frequency of infection, affected limb, type of swelling, fibrotic tissue, range of motion of the affected limb, the location of affected by lymphedema, the degree of lymphedema, the number of treatment sessions (days), and the edema volume.

Also, new variables were added to the data set as predictor variables by reviewing and performing modeling in the relevant software. Due to the difference between the two variables of diagnosis date and date of birth, the patient's age at the time of diagnosis was added to the data set as the "age" variable. The two variables of referral date and date of birth were removed from the data set. In the first 30 repetitions of the proposed method, the correlation of the dominant and involved hand was determined as code zero for alignment of the two and code one for non-alignment. Also, in the following 30 repetitions of the proposed method for this variable, code one was considered for alignment of the two, and code zero for non-alignment. BMI replaced the height, and weight variables from the data set and was added to the data set as an input variable.

Relative indices were used as model input to increase the quality of the model. Therefore, the new variable "LnAff/LnExe" ratio was added to the data set by dividing the number of involved lymph nodes by the number of removed lymph nodes. According to the literature review and the clinician's approval, edema volume greater than 200 mL was considered lymphedema. The variables of class one, representing patients with edema volume more than 200 mL, and class zero, representing patients with an edema volume less than 200 mL, were added to the data set.

#### Optimizing feature selection for each base learner by the genetic algorithm

**Fig. 1:**
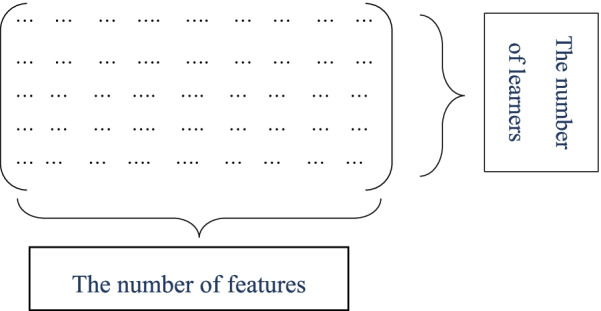
Matrix of solutions with feature selection

This phase's second step was selecting a model feature with a Genetic algorithm. This meta-heuristic algorithm is generally population-based and has a striking global search strategy in an iterative process to find the optimal or near-optimal solution. The search process begins with producing a random primary population of chromosomes in this algorithm. Each chromosome represents a possible solution to the problem, and it is considered a binary string of length N, where N is the total number of initial properties. Suppose attribute "i" is present on the chromosome. In that case, the value of "i" in the corresponding binary string is equal to "1", and if the attribute "i" is omitted in the corresponding binary, the value of "i" is equal to "0". This matrix is shown in Fig. 1.

In each iteration of the Genetic algorithm, there are two general steps. The first step is to assess the suitability of the produced solutions, and the second step is to update the population (new population production). These two sequential steps are performed repeatedly until the termination condition is met. The termination condition in this study was the completion of the algorithm iteration number. Data mining algorithms were used after determining the relevant variables and preprocessing the data, including deleting duplicate data, deleting redundant variables, identifying missing data, reducing the values of variables, and defining new data. The base algorithms of classification, including C5, KNN (with k = 3), SVM (with different kernels Linear, RBF, and Polynomial), LDA, BAYES, and MLP, were independently used in the data section.

#### Optimizing the combined ensemble learning method

The third step was to optimize the combined ensemble learning method and select its features. Instead of using fixed training and testing data sets, this method used the K-fold method. At first, the training dataset was divided into K data folds. After each iteration, the training and test data sets in these K folds differed from the previous iteration of the algorithm. Each classification algorithm's output was considered based on its weight coefficient. The outputs of the classification algorithm—based on their weight coefficient—were considered input data for the Genetic algorithm. Subsequently, the output for each learner was optimized using a Genetic algorithm. The ensemble learning method was used by majority voting extracted by KNN algorithms with K = 3, SVM with kernel RBF, LDA, BAYES, and C5.

Therefore, each chromosome can be considered a solution as Sol, consisting of a vector of length R and a binary matrix R * N, where R is the number of base learners (R = 5), and N is the number of main features. According to Eq. (1), S.EL (j) has continuous values between 0 and 1, indicating the base learners' weight. Also, each line in S.FS represents the attribute selection for each base learner.

1 $${\text{S}}.EL\left( j \right) = w_{j} \in \left[ {0,1} \right];\quad \forall j \in \left\{ {1,2,...,R} \right\}$$

$$S.FS\left( {j,k} \right) = \left\{ {\begin{array}{*{20}c} 1 & {{\text{ if the k attribute exists for the base algorithm}}} \\ 0 & {{\text{otherwise }}} \\ \end{array} } \right.$$

**Fig. 2:**
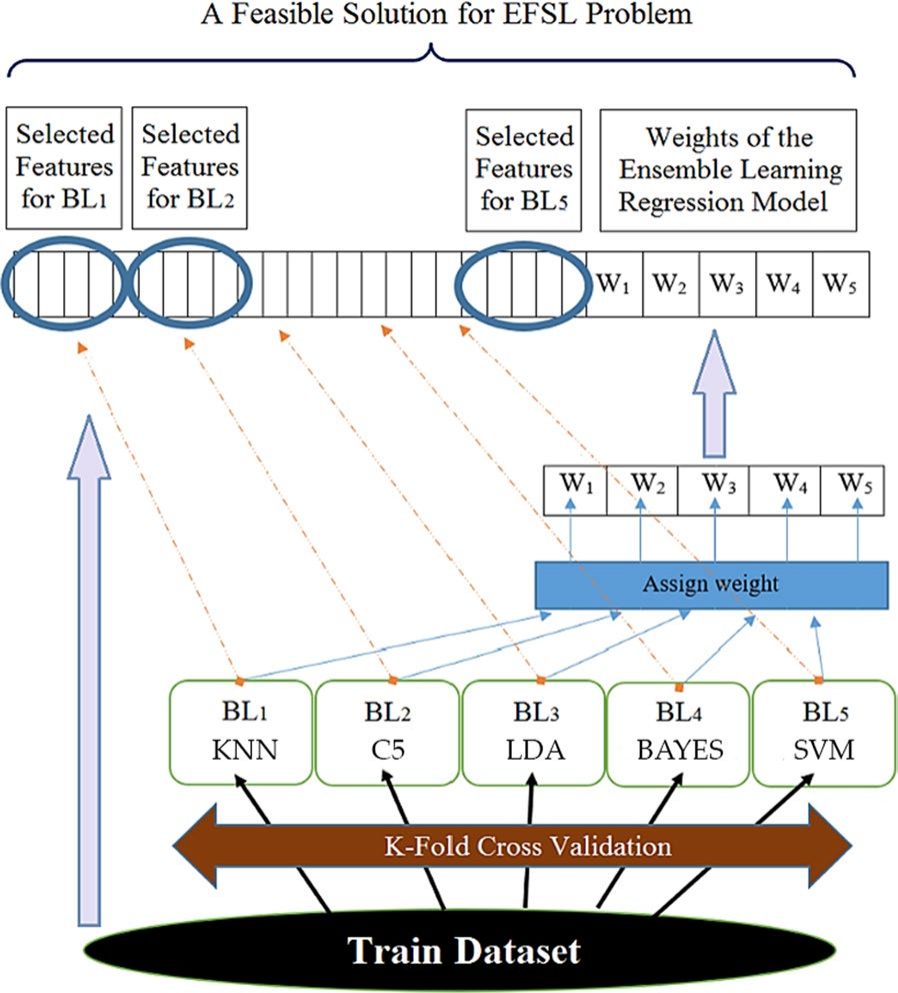
Optimization of the combined method of collective learning and feature extraction

In this method, a chromosome was considered as follows (Fig. 2).

In fact, for each classification, features that were necessarily appropriate for the same classification algorithm were considered. Adjusting the parameters of evolutionary algorithms significantly affects the algorithm's performance. Different parameters and operators were evaluated several times to achieve the appropriate result. Finally, the final parameters and the best operators were selected for the Genetic algorithm (Table 1).

**Table 1:** Adjustment of Genetic algorithm parameters

Parameters description	Values
Number of repeats	100
Population size	50
Percentage of the best current-generation chromosomes (PR)	10
Percentage of combination operator (PC)	60
Percentage of Mutation Operator (PM)	30
Parental selection in selection and jump operator	Roulette Wheel Selection (RWS)

The final model includes five heterogeneous learners for discrete data, including K-Nearest Neighbors (KNN), Support Vector Machine (SVM), LDA, Decision Tree (C5), and BAYES. Each base learner is trained separately under their chosen characteristics. Then, trained models are assembled to determine whether the new patient has lymphedema or not (Eq. (2)). In this equation, w1 to w5 are the weights of the base learners, respectively. The higher the weight value, the more significant the base learner is in the group learning model.

2 $$Out_{i} = \frac{{w_{1} Out_{KNN}^{i} + w_{2} Out_{SVM}^{i} + w_{3} Out_{BAYAS}^{i} + w_{4} Out_{C5}^{i} + w_{5} Out_{LDA}^{i} }}{{\mathop \sum \nolimits_{j = 1}^{5} w_{j} }}$$

Considering the theorem nature and due to the final goal in this part of the project being 0 and 1, we defined the following threshold in Eq. (3).3

**Fig. 3:**
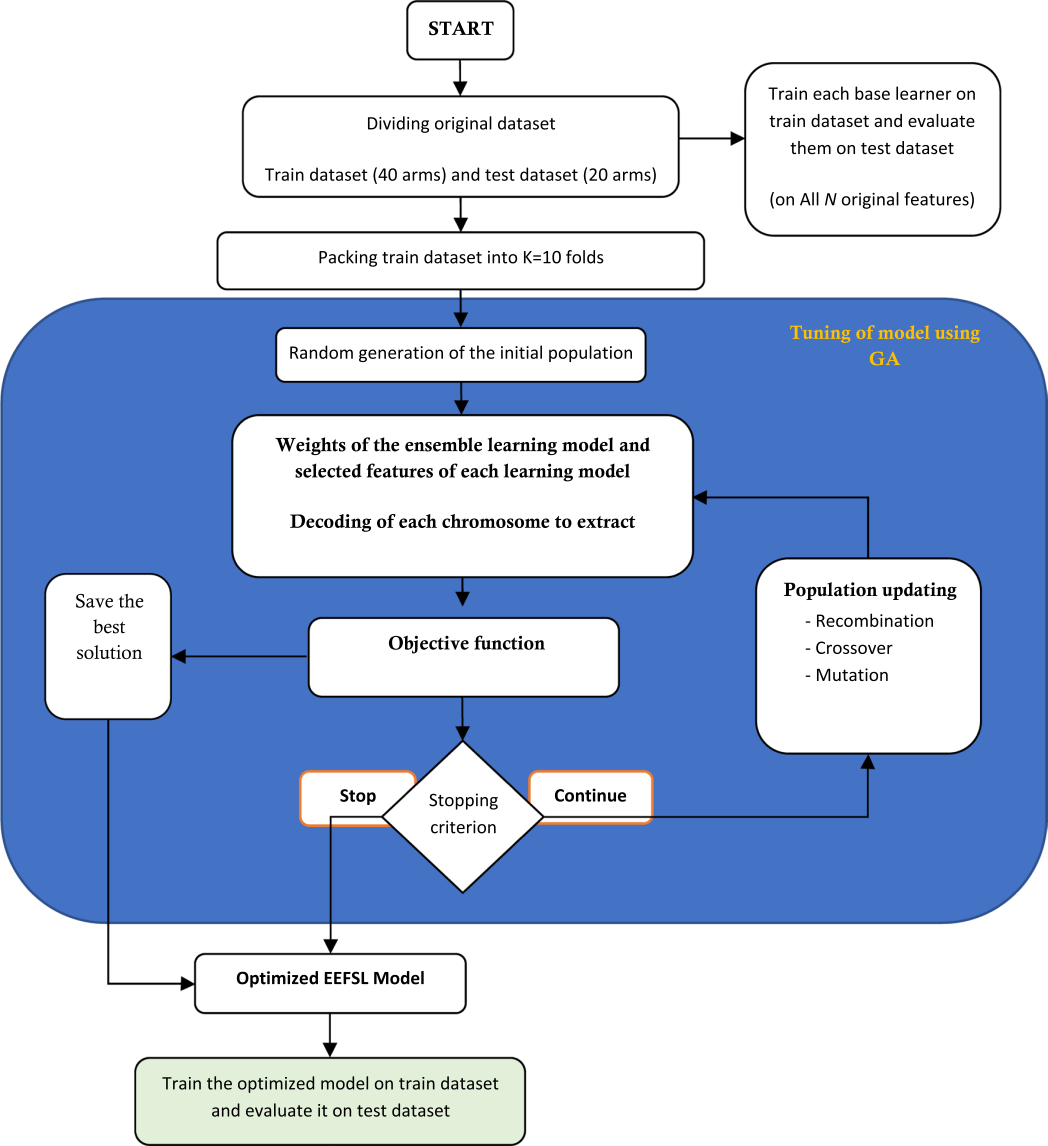
Flowchart of the model used

This study used K-fold algorithms, the optimized feature selection for each base learner, and ensemble learning with the selected classification algorithms to increase the model's accuracy. Finally, the Genetic algorithm was used to improve the output. The flowchart of the model presented is shown in Fig. 3.

#### Estimating fitness function for evaluating appropriate model via the Genetic algorithm

In the phase of identifying the appropriate model to estimate the probability of lymphedema, a three-objective fitness function was determined that was converted into a single-objective function by a weight coefficient to optimize, assess and select the Genetic algorithm. Since different variables are of the same error and percentage, the weight coefficient was defined. If the weight factor is higher, the variable is more important, and if the weight factor is smaller, that variable is less important. Because our data were discrete at this stage, Eq. (4) used for discrete variables in data mining was applied to the output. In this equation, the target class is healthy individuals, and Cost_i_ is the chromosome "i" error.

4 $$Cost_{i} = \min (W_{c1} \times Err_{i} + W_{c2} \times FPR_{i} + W_{c3} \times FRR_{i} )$$

Err, FPR (False Positive Rate), and FRR (False Rejection Rate) are the classification error, error acceptance rate, and error rejection rate, respectively, according to the feature subset of each solution. In this study, a hybrid method called K-Fold Cross Validation has been used to improve the generalizability of the feature selection algorithm. This method considers training and test data differently in the number of K times. Initially, k was considered between 6 and 14. After determining the efficiency, we concluded that the final efficiency increased if k is between 6 and 10. For k equal to 10, we reached the highest efficiency. For k higher than 10, the performance decreased. The whole data was divided into ten parts, and each time nine parts were used for training, and the tenth part was applied for testing.

Finally, the classification error criterion was the mean classification error in 5 different tests. The objective function consists of three parts, in which w_C1, w_C2, and w_C3 are three constant weighting coefficients that regulate the effect of the three error parts on the objective function. The target function was defined as three-variable and adjustable so that the weights could be changed as desired by the clinician. For example, it can be assumed that sick people who are diagnosed as healthy are more important than healthy people who are diagnosed as sick (Wc2 < Wc3). Thus, an objective function is obtained, in which, instead of each chromosome, there is a number for cost. According to the number, it is determined which chromosome is better (Eq. (4)).

After updating the population, the combination operator evaluated the generated solutions in each iteration. The population update in the Genetic algorithm consists of three parts: the direct transfer of a percentage of the best current-generation chromosomes, the combination operator, and the mutation operator. The percentages of next-generation chromosomes are defined as P_Recombination_, P_Crossover,_ and P_Mutation_, respectively, and the sum of these three percent equals 1. In this study, the values of these three coefficients were considered equal to 0.1, 0.5, and 0.4, respectively.

There are several methods for selecting parents in the Genetic algorithm. The roulette wheel selection with a power of 2 was used to determine the parents in the combination and mutation operator. In this method, the most common method of selecting parents in the Genetic algorithm, the probability of choosing each is directly related to the competence of that chromosome (Eq. (5)). In this study, each chromosome competence was assessed by Eq. (6), which Cost is the value of the objective function according to Eq. (4). The lower the error of a solution, or in other words, the more worthy the error, the more likely it is to choose.

5 $$P_{i } = \frac{{ \left( {fitness_{i} } \right)^{ \propto } }}{{\mathop \sum \nolimits_{j = 1}^{population size} \left( {fitness_{j} } \right)^{ \propto } }}$$

6 $$fitness = \frac{ 1}{{Cost_{i} }}$$

In the phase of determining lymphedema risk factors as influential variables in the final algorithm, the average of significant variables in the output was used because of the meta-heuristic nature of algorithms. After averaging the variable in the output, the impact percentage of the variable was determined. The most effective lymphedema risk factors were identified by the final evaluation of the proposed method of influential factors.

### Phase 2: identifying the lymphedema risk factors

To determine the predictors of lymphedema, we considered the final hybrid algorithm as the selected algorithm. Due to the meta-heuristic nature of the Genetic algorithm, the final model was repeated at least 30 times. The effective variables in the output were identified after each repetition. After organizing the number of effective features, we derived these feathers by term of the whole dataset. So, we can ask how much a certain feature affects the output per person. As a result, a lymphedema expert can make a decision for each patient with a certain feature. They can decide more effectively if this feature exists in the effective feature list.

## Results

From 1117 patients, data of 970 patients were examined in the final analysis, and 157 patients were excluded because of incomplete data. Some demographic and clinical features of the patients are reported in Table 2. The mean age was 50.19 (± 11.12) years in patients with lymphedema and 48.00 (± 10.75) years in patients without lymphedema. Most of the patients in both groups were married, homemakers, with a diploma and lower education. The mean number of involved nodes in patients with and without lymphedema was 11.99 and 11.44, respectively.

**Table 2:** Demographic and clinical variables of patients in the study groups

Variable	Group	
	Without lymphedema (n = 230)	With lymphedema (n = 740)
	N (%)	N (%)
*Occupation*		
Housewife	168 (75.7)	573(82)
Employed	54 (24.3)	126 (18)
*Education*		
High school diploma and lower education	131 (58)	499 (70.9)
Higher education	95 (42)	205 (29.1)
*Marital status*		
Single	32 (13.9)	83 (11.4)
Married	192 (83.5)	616 (84.7)
Divorced	4 (1.7)	22 (3)
Widow	2 (0.9)	6 (0.8)
*Type of surgery*		
Mastectomy	120 (52.2)	496 (67.9)
Breast preservation	110 (47.8)	235 (32.1)

	Mean (± SD)	Mean (± SD)
Age	48 (10.75)	50.19 (11.12)
BMI	27.36 (5.1)	28.95 (5.14)
Duration of lymphedema (months)	7.61 (15.97)	22.28 (40.41)
Number of removed lymph nodes	11.44 (6.15)	11.99 (6.04)
Number of involved lymph nodes	2.05 (3.85)	4.09 (5.39)

### Selecting and determining the appropriate model for estimating the risk of lymphedema:

In this step, single classification algorithms were determined by medical and clinical data. Their confusion matrix, FPR, FRR, Accuracy, and Cost evaluation were defined, too (Table 3).

**Table 3:** Evaluation of classification algorithms

Classification			
Evaluation	Confusion Matrix		Algorithm
FPR = 0. 2500	143	547	SVM (Linear)
FRR = 0. 2072			
Accuracy = 0. 7706	555	185	
Cost = 0. 2291			
FPR = 0. 1486	53	637	SVM (RBF)
FRR = 0. 0768			
Accuracy = 0. 8860	630	110	
Cost = 0. 1135			
FPR = 0. 2365	22	668	SVM (Polynomial)
FRR = 0. 3190			
Accuracy = 0. 8622	565	175	
Cost = 0. 1363			
FPR = 0. 2757	142	548	LDA
FRR = 0. 2058			
Accuracy = 0. 7580	536	204	
Cost = 0. 2415			
FPR = 0. 3959	71	619	KNN
FRR = 0. 1029			
Accuracy = 0. 7455	447	293	
Cost = 0. 2525			
FPR = 0. 4514	120	570	Bayes
FRR = 0. 1739			
Accuracy = 0. 6825	406	334	
Cost = 0. 3155			
FPR = 0. 1622	138	552	C5
FRR = 0. 2000			
Accuracy = 0. 8196	620	120	
Cost = 0. 1807			
FPR = 0. 2216	189	501	MLP
FRR = 0. 2739			
Accuracy = 0. 7531	576	164	
Cost = 0. 2472			

**Fig. 4:**
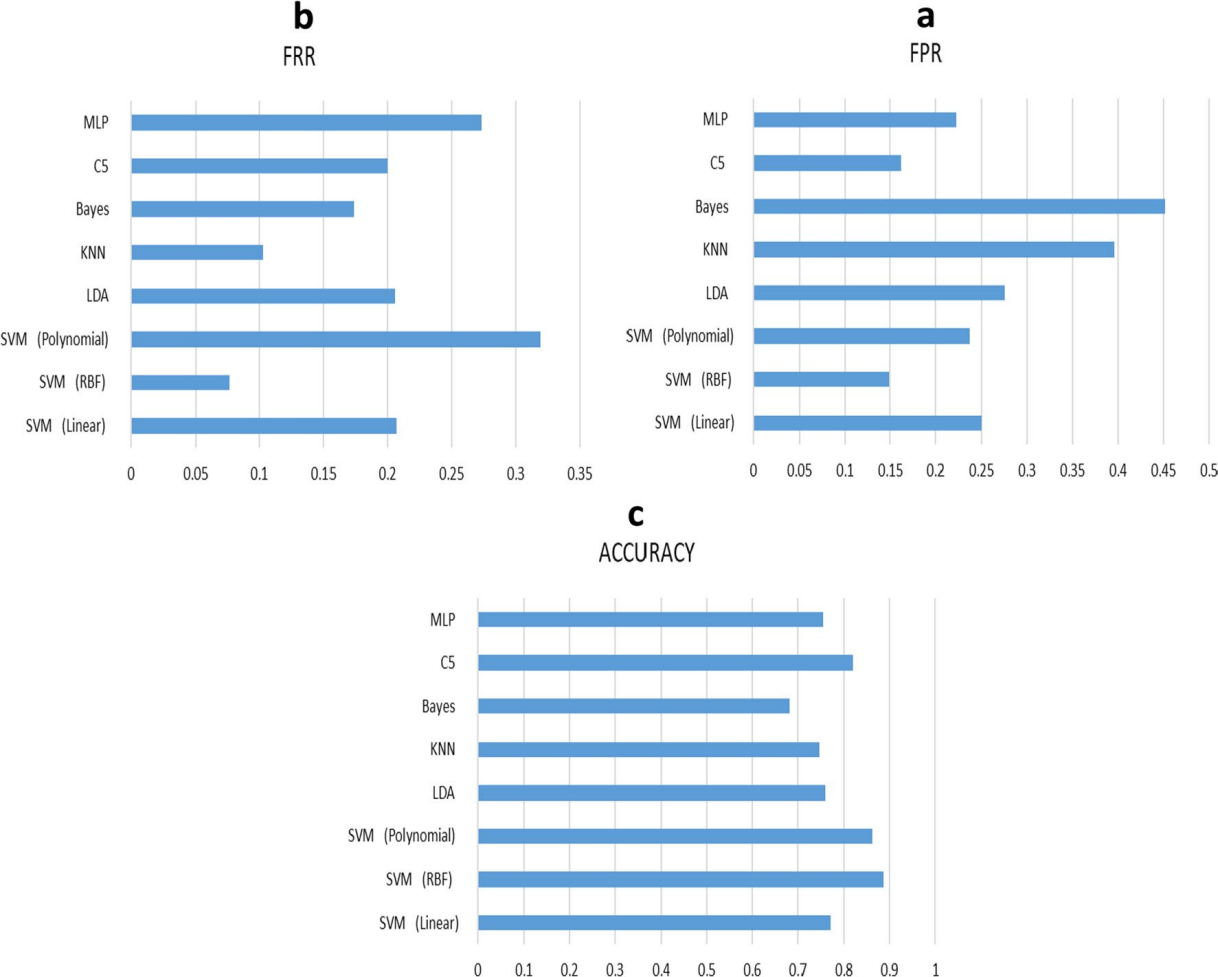
Evaluation **a** FPR, **b** FRR, **c** ACCURACY in classification algorithms

As shown in Table 3, the SVM algorithm with RBF kernel had the best medical and clinical data results in all three evaluations of FPR, FRR, and Accuracy. Based on the Accuracy obtained, this algorithm correctly detects the presence or absence of lymphedema in newly diagnosed patients up to 88% (Fig. 4).

**Fig. 5:**
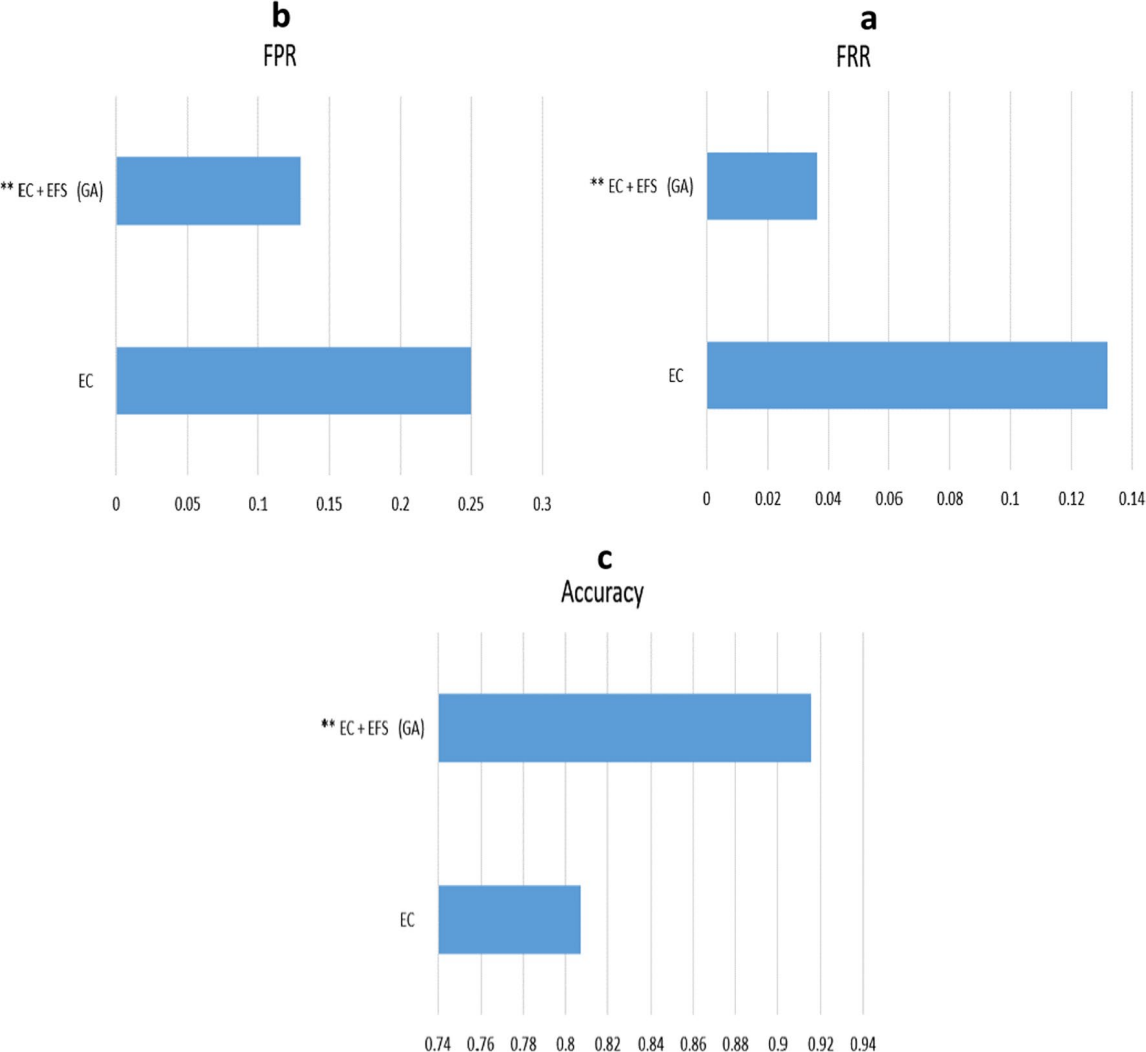
Evaluation of **a** FRR, **b** FPR, **c** ACCURACY in collective learning and suggested methods

Table 4 shows the confusion matrix, FPR, FRR, Accuracy, and Cost evaluation of group learning methods with selected classification algorithms and group learning methods with selected classification algorithms with optimized feature extraction methods. The results indicated that the criteria of FPR (the ratio of negative cases which were detected as positive) and FRR (the ratio of positive cases which were detected as negative) in the proposed ensemble learning method with selected algorithms through the optimized feature selection method had the best evaluation (Fig. 5).

**Table 4:** Evaluation of group learning method with selected algorithms and the proposed method

Name	Evaluation
EC	FPR = 0.2500
	FRR = 0.1319
	Accuracy = 0.8070
	Cost = 0.1922
EC + EFS (GA)	FPR = 0.1297
	FRR = 0.0362
	Accuracy = 0.9154
	Cost = 0.0840

### Determining lymphedema risk factors by the appropriate model:

**Fig. 6:**
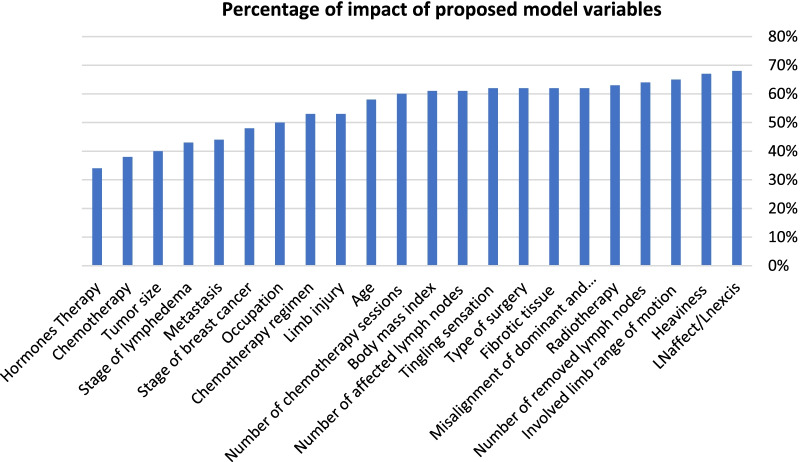
Impact percentage of the proposed model variables in order of impact factor

According to the final evaluation of the proposed model (Fig. 6), the most influential risk factors of lymphedema included the number of involved lymph nodes to the number of removed lymph nodes ratio (68%), feeling of heaviness (67%), involved limb range of motion (65%), number of removed lymph nodes (64%), receiving radiotherapy (63%), non-alignment of dominant and involved limb (62%), fibrotic tissue (62%), type of surgery (62%)), tingling sensation (62%), number of affected lymph nodes (61%), BMI (61%), number of chemotherapy sessions (60%), age (58%), limb injury (53%), chemotherapy regimen (53%), occupation (50%), stage of breast cancer (48%), metastasis (44%), stage of lymphedema (43%), tumor size (40%), receiving chemotherapy (38%) and hormone therapy (34%).

In the first 30 repetitions of the proposed model, if the dominant and involved limb were aligned, the related variable was defined as "0", and if they were non-aligned, it was considered "1". The result of this consideration is equal to 62%. But in the subsequent 30 iterations of the proposed model, this variable was considered "1" if it was consistent and "0" if it was inconsistent. This consideration was 45% effective in determining the result. Therefore, the alignment of the dominant and involved limb was more important.

## Discussion

Our study predicted the probability of developing lymphedema with an appropriate accuracy via the combined method of ensemble learning algorithms and optimized feature selection with the Genetic algorithm. Ensemble learning is a general meta-approach to machine learning that seeks better predictive performance by combining the predictions from multiple models. The three main classes of ensemble learning methods are bagging, stacking, and boosting; we applied the bagging model. We can use the single classification algorithm, but we decided to use five classification algorithms and get the selected features from different algorithms. The results of the ensemble learning algorithm showed that even if the classifiers are significantly weak when combined as a group and executed on data and features with unbalanced distribution, the outcome is acceptable. They make the relevant features by the data sets with plenty of features, limited samples, and unbalanced class distributions [18]. Uneven class distribution occurs when at least one class is not displayed enough and is covered by other classes. The unbalanced data training classification creates many barriers to learning algorithms and offers many implications for real-world applications [19]. Due to this problem, minority class samples are less attended to, affecting incorrect classification results [18]. The classification error of an unbalanced data set is exacerbated by the limited number of samples and a large number of features [20, 21]. Therefore, it is necessary to consider selecting an appropriate analysis model based on such unbalanced data in computer modeling.

The result showed that factors such as number of removed lymph nodes (64%), number of affected lymph nodes (61%), BMI (61%), number of chemotherapy sessions (60%), age (58%), metastasis (44%), and receiving chemotherapy (38%) affected determining lymphedema. A study investigated the influential factors on lymphedema occurrence after the initial treatment of invasive breast carcinoma. The univariate analysis indicated that the obesity category of BMI significantly affected developing lymphoma. There was a significant difference between the mean number of involved lymph nodes in patients with and without lymphoma. Patients' age, tumor location and size, metastasis, chemotherapy, type of surgery, number of removed lymph nodes, and radiation therapy were not associated with lymphedema [22]. Considering the mentioned variables indicative of advanced breast cancer stages, providing lymphedema preventive education for patients in these stages is necessary.

By examining the frequency of age, occupation, and education, we concluded that the mean age of patients with and without lymphedema was 50.19 (± 11.12) and 48 (± 10.75) years. From The people with lymphedema, 82% were housewives, and 70.9% had a high school diploma or lower. A study examining the epidemiological characteristics of patients with breast cancer-related lymphedema showed that 78.5% of patients were over 45 years old, and 53% had been educated for less than 12 years [23]. It appears that housewives or women with a lower education level are at a higher risk of lymphedema, so informing patients about this complication and educating them on preventive strategies, especially in high-risk groups, is recommended.

In this study, the model's accuracy was increased because the data mining algorithms were not used separately, and all the functions of the relevant data mining algorithms were used collectively. Ahmad LG et al. have predicted breast cancer recurrence via the base data mining algorithms like decision trees, support vector machines, and artificial intelligence. Clementine software analysis demonstrated that the extent of lymph node involvement, tumor size, and lymph node inflammation are considered risk factors for breast cancer recurrence [13]. Another study predicted the risk of osteoporosis by three algorithms in data mining, including decision tree CHAID, C5.0, and artificial neural network. They analyzed the data of 671 patients consisting of personal, lifestyle, and disease information and the results of the DEXA device. They extracted the significant features using data mining and its methods. They found that osteoporosis can be better predicted by each algorithm in a particular group of people [24]. Safdari et al. used decision tree and neural network models to collect the data on 351 patients with cardiovascular diseases in 2012. This information was obtained using the Morgan table from the patients referred to Shahid Rajaei Heart Hospital in Tehran. The main objective of this study was to predict the risk of myocardial infarction with a decision tree based on risk factors [12]. Comparing the studies, we conclude that the data mining algorithms with the basic Genetic algorithm can create a high accuracy in our hybrid model. This is because our goal is mainly to build a two-layer collective algorithm (a combination of collective learning algorithms and feature selection). According to the results obtained, each basic algorithm can respond with high reliability.

According to the results and considering our goal, which was to build a two-layer ensemble algorithm or, in other words, to combine ensemble learning algorithms and feature selection, each of the basic algorithms could respond with high reliability. In 2013, Haghighat et al. reported a 30% prevalence of lymphedema by diagnosing lymphedema in 123 of 410 patients with breast cancer at three cancer treatment centers. The patient's mean age in the case and control groups was 50.6 and 84.4 years, respectively. About 41% of the subjects in the case group and 54% in the control group had a high school diploma or higher. The mean BMI, higher grade of the disease, lymph nodes involvement, comorbidities, history of trauma, injury or infection of the limb, number of involved lymph nodes, and time interval from surgery were significantly associated with lymphedema occurrence. The multivariate logistic analysis showed that high BMI, the number of involved lymph nodes, and the time interval from surgery were effective factors correlated with lymphedema [25]. The effect of these factors can be modified and controlled. The logistic model did not fit into our hybrid algorithm because changing the input even with a small number of features did not no significantly change the output. However, this article's simple and combined influential features and our results have a high overlap. A study by Fazeli et al. examined the factors affecting lymphedema using basic algorithms on the same database like ours. They concluded that the support vector machine algorithm with 77.49% accuracy estimates the best performance among C5, C&RT, CHAID, QUEST, SVM, and neural network algorithms [26]. In these similar studies, the algorithms used were basic computer algorithms or improved with genetics and other algorithms. In the current study, the model's accuracy was increased to an acceptable level due to the Genetic algorithm, selective learning, and simultaneous use of selected classification and K-fold algorithms.

Today, with surgical methods and pioneering gland sampling, the prevalence of lymphedema has decreased. Since lymphedema is a progressive and chronic disease, its early detection or prevention is challenging for patients and health professionals. The possibility of its occurrence can be predicted by determining the risk factors of lymphedema, and appropriate strategies can be adopted to moderate some of these risk factors.

Based on the results of a study on Iranian patients, education level, BMI, breast cancer stage, number of lymph nodes involved, comorbidities, hand injury, infection, and duration after surgery were significantly associated with increased lymphedema risk such that an increase of 1 kg / m2 in BMI, one more gland involved and a one-month increase in the period after surgery increased the odds of developing lymphedema by 1.09, 1.15, and 0.01 times, respectively [30]. Although the risk factors obtained from this study are similar to the present study, due to the small sample size, the percentage of the impact of these risk factors on the larger sample size is expected to change. In the present study, the ratio of the number of lymph nodes involved to the number of lymph nodes removed, feeling of heaviness, and range of motion of the affected limb increased by 68%, 67%, and 65%, respectively. Although BMI by 61% was entered after other risk factors, the time after surgery was not recognized as a risk factor via the computer model used. Despite the relative generalizability of the results to patients in other lymphedema centers in the country, it is better to consider the limitations of data recording in interpreting the results.

Lymphedema risk factors are associated with treatment or personal characteristics, adjusted or prevented. According to the results of our study, BMI, as one of the individual characteristics-related factors, increased the risk of lymphedema by up to 61%. In the Ahmed and Dominick study of 1287 and 2431 breast cancer survivors [27, 28], BMI was identified as a significant risk factor for lymphedema. Therefore, self-management education, nutrition counseling, and weight loss can modify one of the most important lymphedema risk factors.

Lymphedema is often diagnosed using subjective and objective measurement tools [29]. Subjective measures consist of self-reported symptoms such as a feeling of heaviness, numbness, or tingling in the patient, which can be addressed using specific lymphedema questionnaires or a list of symptoms [30, 31]. The model in this study predicted 67% and 62% risk of lymphedema occurrence through symptoms of heaviness and tingling in the limb, which can be considered the critical risk factors. A study demonstrated that self-reported symptoms such as heaviness, numbness, and swelling could be the primary predictors of lymphedema [32]. Thus, lymphedema can be detected in the early stages via subjective and objective criteria such as volumetric or circumferential measurements, which improve therapeutic outcomes.

Because of the data set, we could not use logistic regression, but we applied the other classification algorithms as the pack of 2-layer ensemble feature selection. Therefore, we gained an acceptable accuracy. By comparing the performance of five machine learning algorithms in detecting lymphedema, Fu and colleagues reported that the artificial neural network achieved the best performance with an accuracy of 93.75% [9]. Using a group of classification algorithms can lead to the best results for a particular data set. There are different results for each learner, such as the C5.0 algorithm, which can be an acceptable tool for prediction [14]. We simultaneously applied the 2-layer algorithm combined with ensemble learning and feature selection and optimized this combination with the genetic algorithm. By comparing the different classification algorithm methods in the feature selection field, the performance of six classification methods was improved. The results show ensemble learning methods perform better than individual classifications [33]. Therefore, we applied more algorithms in the model and performed better than each individual algorithm alone.

In the first phase of this study, the obtained computer model determined the ratio of the involved lymph nodes and the removed lymph nodes, radiation therapy, type of surgery, and the number of the involved lymph nodes as significant lymphedema risk factors with a probability of higher than 60%. A study on 2431 breast cancer patients showed that lymphedema had developed in 692 participants. Univariate analysis revealed a positive association between lymphedema and mastectomy plus radiation therapy, the removed lymph nodes more than 11, and BMI higher than 25 [34]. Although similar studies have reported the positive association of some demographic and clinical characteristics with the incidence of lymphedema, each factor's contribution to disease development is unclear. The present study determined the risk proportion of each element in percentage by using data mining algorithms and choosing the most accurate algorithm for Iranian women.

Our results identified chemotherapy as a lymphedema risk factor with a risk proportion of 38%. Nguyen et al. showed chemotherapy as a lymphedema risk factor [35], while another study reported no risk proportion for chemotherapy (OR = 1.02, 95% CI = 0.81–1.28) [28]. Variables in different studies have been defined in various classifications, which can explain the disparity in results. For example, Nguyen examined different chemotherapy regimens in regression analysis, and Dominic assessed the impact of receiving chemotherapy on lymphedema. In our study, receiving chemotherapy was included in the final analysis model regardless of the chemotherapy regimen. Its mean was introduced as a lymphedema risk factor after 30 repetitions.

The approved model in this study reported the degree of breast cancer with a 48% probability of effectiveness in causing lymphedema; compared with the study of Ahmed et al., it increased 3.92 times [31]. In a meta-analysis of 98 studies on the risk factors of lymphedema, the authors did not mention the degree of the tumor as a risk factor [35]. In a cross-sectional study of 1974 patients with breast cancer, multivariate analysis of tumor stage was not significantly associated with an increased incidence of lymphedema [34]. Given that the need for more invasive treatments increases as the tumor stage increases, it can be assumed that the potential risk in our study for this factor was a reflection of the use of these treatments. According to the model used in this study, radiotherapy, chemotherapy regimen, and the number of chemotherapy sessions increases the probability of lymphedema by 63%, 53%, and 60%, respectively. It is clear that in the higher stages of the disease, the number of chemotherapy sessions and the type of diet will lead to more sessions and more potent medications.

This study was conducted as one of the first studies in data mining using the method of predicting the risk of lymphedema. Since the model confirmed in the present study has an accuracy coefficient of 91%, the reported risk factors can accurately predict the likelihood of lymphedema. Therefore, physicians and health professionals can design treatment, education, and prevention measures based on each individual, taking into account these risk factors.

## Conclusion

Using data mining algorithms for managing chronic diseases has received much attention. The proposed ensemble learning with selected algorithms with the optimized feature selection with an accuracy of 91% as the most appropriate model could determine the probability of the risk factors of lymphedema in the Iranian female population. Some of the most significant factors introduced in this study were the number of lymph nodes affecting the number of lymph nodes removed, the feeling of heaviness, and the range of motion of the involved limb. The results of this study can be the basis for the use of digital technologies in the management of lymphedema in future studies.

## Data Availability

The datasets generated and analyzed during the current study are available with the corresponding authors: haghighat@acecr.ac.ir, sha_haghighat@yahoo.com.
